# Validity of mobile electronic data capture in clinical studies: a pilot study in a pediatric population

**DOI:** 10.1186/s12874-017-0438-x

**Published:** 2017-12-08

**Authors:** Belinda von Niederhäusern, Ramon Saccilotto, Sabine Schädelin, Victoria Ziesenitz, Pascal Benkert, Marie-Luise Decker, Anya Hammann, Julia Bielicki, Marc Pfister, Christiane Pauli-Magnus

**Affiliations:** 1 Clinical Trial Unit, Department of Clinical Research, University and University Hospital of Basel, Schanzenstrasse 55, 4031 Basel, Switzerland; 2 Department of Clinical Research, University and University Hospital of Basel, 4031 Basel, Switzerland; 3 Division of Paediatric Pharmacology & Pharmacometrics, University of Basel Children’s Hospital, 4031 Basel, Switzerland; 4 Department of Paediatric Infectious Diseases, University of Basel Children’s Hospital, 4031 Basel, Switzerland

**Keywords:** Remote studies, Mobile studies, Pediatrics, Data validity, Data quality, Feasibility

## Abstract

**Background:**

Clinical studies in children are necessary yet conducting multiple visits at study centers remains challenging. The success of “care-at-home” initiatives and remote clinical trials suggests their potential to facilitate conduct of pediatric studies. This pilot aimed to study the feasibility of remotely collecting valid (i.e. complete and correct) saliva samples and clinical data utilizing mobile technology.

**Methods:**

Single-center, prospective pilot study in children undergoing elective tonsillectomy at the University of Basel Children’s Hospital. Data on pain scores and concomitant medication and saliva samples were collected by caregivers on two to four inpatient study days and on three consecutive study days at home. A tailored mobile application developed for this study supported data collection. The primary endpoint was the proportion of complete and correct caregiver-collected data (pain scale) and saliva samples in the at-home setting. Secondary endpoints included the proportion of complete and correct saliva samples in the inpatient setting, subjective feasibility for caregivers, and study cost.

**Results:**

A total number of 23 children were included in the study of which 17 children, median age 6.0 years (IQR 5.0, 7.4), completed the study. During the at-home phase, 71.9% [CI = 64.4, 78.6] of all caregiver-collected pain assessments and 53.9% [CI = 44.2, 63.4] of all saliva samples were complete and correct. Overall, 64.7% [CI = 58.7, 70.4] of all data collected by caregivers at home was complete and correct. The predominant reason for incorrectness of data was adherence to the timing of predefined patient actions. Participating caregivers reported high levels of satisfaction and willingness to participate in similar trials in the future. Study costs for a potential sample size of 100 patients were calculated to be 20% lower for the at-home than for a traditional in-patient study setting.

**Conclusions:**

Mobile device supported studies conducted at home may provide a cost-effective approach to facilitate conduct of clinical studies in children. Given findings in this pilot study, data collection at home may focus on electronic data capture rather than biological sampling.

**Electronic supplementary material:**

The online version of this article (10.1186/s12874-017-0438-x) contains supplementary material, which is available to authorized users.

## Background

High quality research relies on the collection of high quality data. Traditionally, this is done in the inpatient setting or through ambulatory visits to a study site, which can present a barrier to participation (e.g. cost, travel burden, time) and a risk to the validity of research (e.g. high loss-to-follow-up, low external validity) [1, 2]. The widespread availability of new technologies has the potential of shifting some research activities, including enrollment, managing trial activity, reporting results, and safety oversight, away from study sites. Such “remote” research may encourage the participation of a more diverse group of patients in research with improved recruitment rates and at lower costs than those of conventional trials [3–5]. A combinatory approach including direct interactions with the study team may allow remote data collection to be optimally leveraged [6, 7], e.g. by addressing challenges around data quality and retention [8–11].

In pediatric care, empirical evidence on the optimal dosing and action of routinely used medicine remains limited [12–14]. The relationship between drug exposure and its effects are often different in children compared to adults. For this reason a simple extrapolation of pediatric dosing based on adult data can put children at increased risk of adverse events and therapeutic failures [15–17]. Therefore, innovative clinical study designs in pediatrics are urgently needed. Currently, major challenges of designing and conducting clinical trials in children, include (i) small sample sizes of pediatric studies, (ii) increased study complexity due to multiple age groups, (iii) integration of research in daily activities of the whole family affecting parental time of work and supervision of other children, and (iv) child absence of routine activities. Together with the burdens of travel and frequent site visits, these limits are associated with low recruitment and high dropout rates [18, 19].

Recent “care-at-home”-initiatives indicate that mobile or remote approaches in clinical research with children have the potential to increase patient and caregiver satisfaction without increasing privately borne costs [20]. In addition, today’s parents and their children are technology savvy and frequent users of mobile devices, suggesting their high potential as future candidates in remote clinical trials. While the methodology is still in its infancy, increasing interest and support from regulators, sponsors, and patients will likely propel remote trials forward in the near future [21]. Thus, further investigation into the methodology of studies that use a combinatory approach is warranted. In this pilot study, we aimed to investigate the feasibility of remotely collecting valid (i.e. complete and correct) clinical data and saliva samples in a pediatric population utilizing mobile technologies. In addition, we assessed the general acceptance, reasons for non-consent, and the resulting costs of this study.

## Methods

### Study design

This was an investigator-initiated, single center, prospective pilot study investigating the feasibility of remotely conducting clinical studies with parents/legal representatives (“caregivers”) and children. We developed a custom mobile application (“app”) allowing patients and their caregivers to participate in the study remotely after an initial training session at our institution (details of application development may be found in Additional file 1). We elected pain management after tonsillectomy as a model due to the frequency of the surgical procedure in children younger than 15 years [22] requiring standardized analgesic therapy and the potential to remotely assess pain levels by caregivers using a validated scale (The Childhood Discomfort and Pain Scale) [23]. In addition, remotely collect saliva samples were planned to measure acetaminophen concentrations mimicking the design of a pharmacokinetic (PK) study. In addition, the local standard of care sequence after tonsillectomy consisting of two to four days inpatient care after surgery allowed study staff to train caregivers in the use of study technologies and procedures for the at-home phase.

Total study duration for each participant and caregivers was 10 days during which data and samples were collected on 2–4 days as an inpatient and on 3 days at home. On day 8, the caregivers filled out a feasibility questionnaire. On day 10, study staff additionally contacted caregivers for feedback on the study in a follow-up telephone interview.

### Participant eligibility

Children presenting for elective tonsillectomy were screened and enrolled at the University of Basel Children’s Hospital from May 26 2016 until January 07 2017, during the pre-surgery anesthetics consultation. Inclusion criteria were age between 2 and 10 years, routine elective tonsillectomy (with or without other additional Ear, Nose, and Throat intervention), anticipated inpatient stay of a minimum of 2 days, willingness and ability of caregivers to understand and implement study procedures in the hospital and at home, and ability of caregivers to understand, speak, and read German. Exclusion criteria were contraindications to acetaminophen administration and any reasons precluding the collection of saliva samples.

### Study procedures

Screening of eligible patients was performed at the pre-anaesthetics clinic consultation by a physician and a study nurse. After assessment of inclusion/ exclusion criteria and written informed consent, the study nurse informed caregivers about the mobile study application, provided them with an instruction manual, and supported them in the setup of their login. Caregivers had the possibility to choose between their own mobile phone (Bring Your Own Device, “BYOD”), or an iPod-Touch provided by the study team for the duration of the study.

**Fig. 1:**
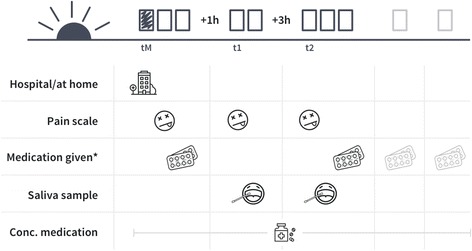
Daily data collection schedule. tM: Timepoint directly after awakening of child, t1: 1 h (+/− 15 min) after administration of first routinely scheduled dose of acetaminophen; t2: 4 h (+2 h) after administration of first routinely scheduled dose of acetaminophen. * “Medication given” indicates the timepoint at which children had either received routine acetaminophen, or not (yes/no). Independent of whether medication was given or not, the app used the recorded time stamp to automatically calculate t1 and t2

On the day of tonsillectomy, a study nurse explained the procedures, data collection and the Childhood Discomfort and Pain Scale to participating children and caregivers. After the surgeon performed tonsillectomy, postoperative pain management with acetaminophen was initiated according to the current standard of care of the hospital [24]. The study did not interfere with routine pain management. Participating children stayed on the ward for approximately 3 days (Fig. 1).

Daily data and sample collection was scheduled to mimic a pharmacokinetics- and dynamics (PK/PD) study. The mobile app issued automatic electronic reminders for scheduled doses as well as pain assessment/sample collection time points. Data entry was automatically time stamped. Pain assessment was repeated 3 times a day by the caregivers upon awakening of the participating children in the morning (tM) and 1 (+/− 15 min, t1) and 4 (+2 h, t2) hours after administration of the first routinely scheduled dose of acetaminophen. Saliva samples were collected twice daily for each participant at t1 and t2. Sample codes were either scanned using the mobile application scanning function or typed in by caregivers. All concomitant medication allowed according to the guidelines of the hospital was documented throughout the study by taking a photograph of the blister or package using the mobile application. After initial supervision by a study nurse, caregivers conducted these assessments autonomously while still in the inpatient setting.

On day 3(+/− 1 day) post-surgery, participating children were discharged home. The study nurse explained how to collect and store saliva samples at home and provided labeled containers for all samples. Children staying in hospital longer than 4 days were excluded from the study. On the 3 days following discharge, caregivers collected data and samples at home following the scheme established during the inpatient stay. On study day 8, a bicycle messenger collected all saliva samples. The mobile application reminded caregivers to fill in a feasibility questionnaire before uploading data to the study server. On day 10, a study nurse conducted a follow-up telephone call for general caregiver feedback.

### Statistical analyses

#### Primary and secondary endpoints

The primary endpoint of the study was the proportion of simultaneously *complete* and *correct* data (pain scale or saliva) in the at-home setting. A complete data set consisted of five data points (three pain scale assessments and two saliva samples) for each day and patient. Complete pain scale data were considered correct if collected within the predefined timeframes (1 h (+/− 15 min) and 4 h (+2 h) after first medication). Complete saliva samples were considered correct if i) collected within predefined timeframe, ii) saliva volume sufficient for potential laboratory analyses, and iii) unique sample ID entered into mobile application. In the initial analysis plan, we aimed to measure acetaminophen levels which were omitted due to technical limitations in reliably detecting acetaminophen in saliva. Secondary endpoints included the proportion of complete and correct samples in the inpatient setting (training setting before hospital release, collected as described for the out-patient setting), the subjective feasibility for caregivers as measured by an electronic questionnaire on day 8, the percentage of consenting patients, the reasons for non-consent, the legibility of photos of concomitant medication using the mobile application, and study cost.

#### Primary analysis

The full analysis set consisted of all patients and caregivers who fulfilled all inclusion criteria and consented to take part. We descriptively summarized the proportion of complete and correct clinical data (pain scale) and samples (saliva) collected in the at home setting. In addition, the number of complete and correct samples per patient was modeled in a logistic regression and the 95% confidence interval (CI) was estimated based on profiled log-likelihood functions. Based on our experience with paper-based patient-reported outcomes (e.g. questionnaires), our hypothesis was that the overall completeness and correctness of electronically collected data would be above a predefined threshold of 90%.

#### Secondary analyses

The proportion of simultaneously *complete* and *correct* data (pain scale or saliva) in the inpatient setting was analyzed as described for the primary endpoint. In addition, both analyses were repeated with a secondary analysis set consisting of 15 patients who used the newest version of the mobile application. Baseline characteristics, study flow statistics, reasons for non-consent, feasibility questionnaires, and legibility of images were summarized and presented descriptively.

#### Missing data and drop-outs

Missing data were part of the primary endpoint (completeness not reached). Patients who were re-hospitalized within three days after discharge were considered drop-outs, and reasons were documented. All other data were assumed to be missing at random and no imputations were performed.

### Cost analysis

We describe a total study cost approach factoring in app development and testing, on-site, data management, analysis staff time, study-specific materials, laboratory sample analysis, and transport costs. Cost calculations were based on salaried staff time log sheets and fixed costs for materials. Sensitivity analyses include cost for a traditional, fully on-site conducted scenario, and cost for larger samples size studies.

## Results

### Patient and caregiver characteristics

**Fig. 2:**
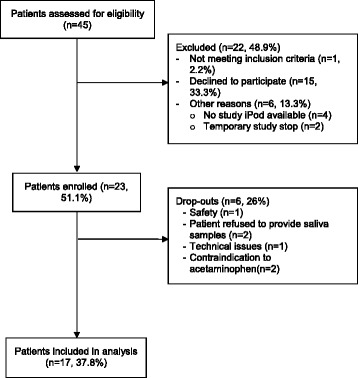
TOMACHI Study Flow Diagram

Of the 45 patients and their caregivers assessed for eligibility, twenty-three (51.1%) were enrolled in the study, and 17 (37.8%) completed the full study (Fig. 2). Of the 15 (33.3%) caregivers who declined to participate, thirteen consented to provide a reason for non-consent, which predominantly included the perceived time burden of the study (8/13, 61.5%) (Table 1). Of 23 enrolled patients, 6 (26%) dropped out during study conduct. Reasons included one non study-related serious adverse event, patient refusal to provide saliva samples, technical issues with the mobile application, and contraindications to acetaminophen (Fig. 2).

**Table 1:** Reasons for caregiver non-consent

	n (%)
Caregivers who declined to participate (*n* = 15) and provided reason for non-consent	13 (86.7)
I do not have the time to conduct the study	8 (61.5)
I do not believe I can collect data and samples correctly	4 (30.8)
I do not want to put additional burden on my child	3 (23.1)
I did not fully understand what the study is about	2 (15.4)
I generally have doubts about clinical research	0 (0)
I would be interested to participate in such a study in the future	6 (46.2)

Baseline characteristics of the 17 patients and caregivers who completed the study are described in Table 2. Median age of patients and caregivers was 6.0 (Interquartile Range (IQR) 5.0–7.4) and 35.0 (IQR 32.0–38.0), respectively. A majority of patients (11/17, 64.7%) had one sibling, and 14/17 caregivers (82.4%) were native German speakers.

**Table 2:** Baseline characteristics of patients and caregivers

n (%)	17	(100)
Patient gender (n male (%))	10	(58.8)
Age (median years [IQR])	6.0	[5.0, 7.4]
Number of siblings (%)		
0	2	(11.8)
1	11	(64.7)
2	2	(11.8)
3	2	(11.8)
Caregiver age (median years [IQR])	35.0	[32.0, 38.0]
Caregiver native German speaker = yes (%)	14	(82.4)
Caregiver working at the moment = yes (%)	12	(70.6)
Caregiver occupation (ISCO) (%)		
At home/unemployed	5	(29.4)
Professional	3	(17.6)
Service and sales workers	9	(52.9)
Caregiver volume of work (median weekly % [IQR])	60.0	[40.0, 70.0]

*IQR* Interquartile Range, *ISCO* International Standard Classification of Occupations

### Completeness and correctness of saliva sampling and pain assessments at home

In total, caregivers collected 303 pain scale assessments and 202 saliva samples. During the at-home phase, 71.9% [CI = 64.4, 78.6] of all pain assessments were complete and correct (92.2% complete, and thereof 78.0% correct) compared to 53.9% [CI = 44.2, 63.4] (77.5% complete, and thereof 69.6% correct) of all saliva samples (Table 3). Overall, 64.7% [CI = 58.7, 70.4] of all data collected by caregivers at home was complete and correct.

**Table 3:** Completeness and correctness of caregiver collected data and samples

			Complete and correct			
			No		Yes	
Location	Item	Total n	n	%	n	%
At home	Pain scale	153	43	28.1	110	71.9
	Saliva samples	102	47	46.1	55	53.9
	All	255	90	35.3	165	64.7
Inpatient	Pain scale	150	57	38.0	93	62.0
	Saliva samples	100	61	61.0	39	39.0
	All	250	118	47.2	132	52.8
All	Pain scale	303	100	33.0	203	67.0
	Saliva samples	202	108	53.5	94	46.5
	All	505	208	41.2	297	58.8

### Completeness and correctness of saliva sampling and pain assessments in the inpatient setting

In the inpatient setting, 62.0% of all pain measurements were complete and correct ([CI = 54.1, 69.5], 94.0% complete and thereof 66.0% correct, respectively) compared to 39.0% ([CI = 29.8, 48.7], 77.0% complete, and thereof 50.6% correct, respectively) of saliva samples. Overall, 52.8% [CI = 46.6, 58.9] of all data collected by caregivers in the inpatient setting was complete and correct.

### Reasons for incompleteness and incorrectness and exploratory sensitivity analyses

Incompleteness of data was mostly due to technical issues which two caregivers experienced (predominantly affecting saliva samples) or the discontinuation of data collection by one caregiver at home. Exploratory analyses of the subgroup of participants who did not experience technical issues with an early version of the mobile application (*n* = 15) showed that completeness and correctness of pain assessments remained the same (71.9%), but that the percentage of correct and complete saliva samples increased from 53.9% to 61.1%. The reason for this was a programming issue, which affected the entry of saliva sample IDs in the application.

The major reason for incorrectness of data was incorrect timing (i.e. data was not collected within predefined timeframes of 1 h (+/−15 min), and four hours (+2 h) after first medication, in Additional file 2: Table S1). Exploratory sensitivity analyses assuming that all data had to be collected within one calendar day instead of the narrow timeframes of 1 and 4 h showed that in this case 92.2% of pain scale data and 74.5% of saliva samples would have been complete and correct at home, and 94.0% and 73.0% in the inpatient setting, respectively (Additional file 2: Table S2). Further, a positive trend for complete and correct data and sample collection was observable from day one in the inpatient setting to day three, i.e. the day of hospital release. On the first day at home, the proportion of complete and correct data increased once more, before then declined slightly (Additional file 3: Figure S1). No clear trend in the quality of data collection could be identified regarding the individual patient and caregiver (Additional file 4: Figure S2), or when stratifying the analysis by number of siblings, caregiver occupation, or caregiver native language.

### Legibility of concomitant medication images

Of the 17 patients who completed the study, 10 (58.8%) took 24 different images of medications using the mobile application’s imaging function. All 24 images were sharp and legible. Medication names were identifiable on 18 (75%) and dosage information (e.g. on drug containers, blisters, etc.) on 13 (54.2%) of all 24 images, respectively.

### Feasibility and practicability for caregivers

Out of 17 caregivers, 15 provided answers in the feasibility questionnaire. Nine of 15 (60.0%) caregivers thought studies at home are a good idea and 53.3% (8/15) would probably take part again (Additional file 2: Table S3). Over 66% (10/15) of caregivers spent less than 15 min on study procedures. In 73.3% (11/15), the mother was the primary caregiver collecting data and samples for the study at home. 60% (9/15) of caregivers said that study goals were explained “very well” to them in the beginning, compared to 53% (8/15) who said study procedures were explained “well”. Usability of the study app was rated between “ok” (8/15, 53.3%) and “great” (6/15, 40.0%). 66.7% (10/15) rated the study procedures “easy” in the inpatient setting compared to 40.0% (6/15) in the at home setting. Asking caregivers about potential difficulties, 86.6% (13/15) answered that the study flow, i.e. the timing of data and sample collection, was sometimes difficult to follow (Additional file 2: Table S4).

### Study cost and cost comparison

Total study cost included fixed costs ($44′577) such as application development and support ($11′955), study-specific materials (two iPods, saliva sampling tubes, envelopes, cafeteria vouchers for caregivers, $2′507), laboratory sample analysis ($2′224), study-material transport costs from caregiver’s home at the end of the study ($474), database setup, management and statistical analysis ($21′360), study monitoring ($6′057) and variable salaried on site staff cost (part time; one physician, three study nurses) over the eight months the study was active ($ 19′157), summing to a total of $63′734 and $3′749 per patient who completed the study.

Our sensitivity analyses for a traditional, hospital-based approach for the same study with the same duration, but six full study days in the inpatient setting and data collection by study nurses suggested total fixed costs of $35′593 compared with $44′577 for the pilot study, and variable on-site staff costs of $20′202 compared with $19′157 for the mobile study summing to a total of $55′795 and $3′281 per patient. The difference was driven by the high initial cost for app development and support, but lower study nursing, physician, and data entry time compared to a traditional trial. Increasing the sample size from 17 to hypothetical 100 evaluable patients would have resulted in cost per patient of $1′077 for the mobile trial and $1′307 for the hospital-based approach.

## Discussion

Results from our pilot study indicate that mobile data and sample collection for clinical studies with children and their caregivers are feasible, yet subject to certain caveats. We were able to engage and enrol patients and to conduct the study with retention rates comparable to those of studies done in traditional settings. Furthermore, the participating caregivers reported high levels of satisfaction and willingness to participate in similar trials in the future. However, the overall proportion of complete and correct data collected in the current framework would not be sufficient to obtain valid study results for a stand-alone PK study. However, sparse PK data collected in such at-home study may be combined with data from more conventional PK studies to enhance PK/PD analyses including pharmacometric modelling. While 92.2% of pain scale data and 77.5% of samples were complete, only 78.0% and 69.6% thereof were correct, respectively. We could therefore not prove our hypothesis of 90% complete and correct data and sample collection in the at home setting. Reasons for this included the narrow timeframe in which data and samples had to be collected by caregivers, the handling of saliva samples, and technical issues with the application.

Expanding the narrow timeframes to one full calendar day, however, would have resulted in over 92% of pain measurements to be complete and correct in the at home setting. We therefore believe that other study types such as phase III or IV studies or observational research with less time-critical data to be collected may be viable options to make use of mobile data collection. Examples may include postoperative observations, the assessment of quality of life outcomes, medication management in chronic conditions or continuous physiological measures using sensor devices.

Exploratory analysis of factors such as number of siblings, caregiver occupation, or caregiver native language did not reveal any clear trend in supporting complete and correct data collection among caregivers. As expected, this pilot study did not yet prove cost-effective due to the development cost of the application. However, future studies including larger samples sizes and building on an improved framework of the existing application will be a cost-efficient option.

Although our participants seemed broadly similar to those in comparable traditional trials, the requirement for ease in mobile phone handling (in our pilot restricted to iPhone and iPods), understanding of the German language, and the active choice by caregivers to conduct the study at home probably may have resulted in selection bias. While this study planned to leverage study participants’ own Internet-enabled mobile devices for remote data collection (“Bring Your Own Device”), we also provided study mobile devices in order to avoid additional caregiver selection bias. External validity is also a common problem for traditional trials, and adequate description of the setting and the sample characteristics is needed. In future mobile trials, we therefore aim to extend the pilot setting to different mobile technologies (i.e. Android) and multiple languages.

Furthermore, the mobile application was designed with the highest user flexibility and –usability in mind in order to minimize user fatigue or dropout. This flexibility in data entry resulted in data points which were often ambiguous (e.g. inconsistencies in automatic time stamp versus time point indicated by caregiver, or typing errors for sample codes). We decided to strictly analyse the data as transmitted by the caregivers although some of these data point ambiguities could have been resolved by the investigators by applying logical cross-checks.

While this pilot was efficient with respect to direct caregiver-reported data entry and on-site staffing levels, data management and analysis of the app-collected data structure was more resource intense than expected. Particular hurdles were (i) the translation of structured data recorded by the mobile application (json-format) into a tabular format suitable for statistical analysis and (ii) to incorporate the flexibility given to the users for data entry. For future studies, translation of structured data can be easily optimized by establishing standardized procedures, whereas specific care must be taken to reduce flexibility in data entry to avoid a high degree of complexity in data analysis.

According to the US Food and Drug Administration, electronic capture of clinical trial source data is nowadays preferred over paper-based data collection [21, 25]. However, data quality has been reported to be problematic [8, 9], and combinations of mobile technologies with appropriate interactive guidance by study staff were suggested to be more successful [3, 4]. To our knowledge, this is the first study explicitly evaluating the quality of data resulting from mobile data capture in a setting imitating pediatric PK/PD modelling. We combined an initial caregiver training session in the inpatient setting including the possibility to interact with study staff with an independent at-home phase. Caregivers were satisfied with study staff support in the inpatient setting, but reported more difficulties following the study procedures at home (Additional file 2: Table S3).

As described by Murray [9] and Coons [25], there are generally two concerns about data quality in clinical studies: Validity – to what extent is the information provided by participants is “true”- and amount of missing data, in terms of item nonresponse. While missing data is generally expected to be minimized with automatic reminders issued by electronic applications, patient-reported data validity may be problematic in both mobile and traditional on paper studies. In this study, we confirm that missing data is less of an issue than data validity. If we had allowed caregivers to collect data and samples within one calendar day rather than within narrow timeframes, 92.2% of all pain measurements and 74.5% of saliva samples would have been complete and correct in the at home setting (Additional file 2: Table S2).

Further, self-reported patient outcomes have generally been criticized before with respect to internal validity and data quality, but are often necessary to adequately evaluate the treatment benefit provided by new interventions [25]. We used a standardised scale for pain assessment [23] by caregivers as a model for mobile data collection. Automatic time stamps for data entry aimed to improve data validity (e.g. correct timing), and allowed the study team to better judge the validity of patient-reported measurements compared to paper based data. In future versions of the application, all patient-reported assessments will therefore be validated for computer use if possible, and methods for case confirmations by study staff will be included to be able to judge the validity of data points.

A recent survey [10] across pharmaceutical companies revealed that just 37% are currently using mobile technologies in clinical trials, of which more than two-thirds (68%) are mobile apps. The primary benefit that companies see for adopting “mHealth” technologies is real-time data acquisition (36%), followed closely by increased patient compliance (30%) and improved data quality (25%). The first successfully completed fully remote Diabetes management trial, the VERKKO trial, sponsored by Sanofi reported initial results on high patient satisfaction rates, reduced study coordination activities, faster study completion, and increased patient retention rates [26]. Nevertheless, companies still have a number of concerns around the technology. Data security is the primary concern for almost a third of respondents (32%), whilst difficulty in incorporation (29%) and resistance from patients or physicians (23%) are both considerable worries. While we did not experience any of these, our study revealed that data quality may only be improved if the mobile application is supporting data collection that is a) well-structured and easy to follow for patients, b) flexible to some extent (i.e. large timeframes), but still rigid enough to assure resulting data quality (i.e. using automatic time stamps rather than patient-reported time points which are prone to error), and ideally, c) remotely monitored. Compared to traditional on paper study settings, users of mobile technologies for clinical studies should make use of their potential of real-life data monitoring and automatic time stamps that should ultimately improve overall patient-reported data validity.

Due to the particular scientific and technological issues associated with the use of mobile devices that currently inhibit their widespread use, the Clinical Trials Transformation Initiative (CTTI) is developing recommendations for managing mobile devices in clinical trials, and guiding principles to promote their inclusion [11].

## Conclusion

In conclusion, mobile studies conducted at home are a feasible approach when certain circumstances are met. Future electronic at-home data collection should predominantly include data that is not time critical at pre-defined, yet flexible time points. Further, the collection, storage, and shipping of biological samples have proven more difficult and should be kept at a minimum. Therefore, we would refrain from conducting remote “stand alone PK studies” that require a rigid data and samples collection schedule. However, studies with more flexible data acquisition schedules, such as clinical phase III or IV trials with sparse PK and biomarker sampling, and observational studies could profit from such an approach. Importantly, mobile applications need to be designed in a well-balanced manner, allowing for user flexibility but also assuring resulting data quality. Current efforts at the CTTI specifically target the technical and scientific issues that still remain with mobile devices in clinical research. In the future, we expect an enhanced version of our current technology to reach wider patient populations and to incorporate lessons learned with features such as remote patient monitoring using real life data capture and increased site-patient interaction.

## Additional files

Additional file 1: Development of mobile application. (PDF 74 kb)

Additional file 2: Table S1. Timing of complete data and samples, by timepoint and location. **Table S2.** Complete and correct data and samples when timing as a correctness factor is neglected (i.e. all data collected within one calendar day are correct). **Table S3.** Caregiver feasibility questionnaire. **Table S4.** Main difficulties experienced by caregivers during conduct of study. (PDF 205 kb)

Additional file 3: Figure S1. Proportion of complete and correct data and samples by location and day. (PDF 6 kb)

Additional file 4: Figure S2. Proportion of complete and correct data and samples by patient and location. (PDF 8 kb)

## Abbreviations

CI: Confidence interval
CTTI: Clinical Trials Transformation Initiative
IQR: Interquartile range
ISCO: International standard classification of occupations
PK/PD: Pharmacokinetic / pharmacodynamic study
