# Supplementary material for: Validity of mobile electronic data capture in clinical studies: a pilot study in a pediatric population
Source: BMC Med Res Methodol. 2017 Dec 8;17:163. doi: 10.1186/s12874-017-0438-x (PMC5721383; doi:10.1186/s12874-017-0438-x)
Supplement: Supplementary file 1 — Development of mobile application. (PDF 74 kb) [file 12874_2017_438_MOESM1_ESM.pdf]

Development of mobile application

A custom mobile application ("app") was developed to allow participants to collect study information in their daily routine as easily as possible. The planned study workflow was extensively discussed with the study team and respective assumptions about the ideal app flow and different input methods were explored using multiple paper-based interactive prototypes [1]. Subsequently, a high fidelity app design mockup [2] was created in agreement with the communication department of the involved hospital. User interaction was tested again internally using clickable design prototypes [3].

Due to the required functionality, the application was developed as native mobile app primarily focused on iOS devices [4-6]. The mobile app was then beta-tested in multiple cycles with various members of the study team and people not directly involved in the study. Finally, the application was made available to the study participants through download from the Apple App Store using the participants' own iOS device or as a pre-installed app on a dedicated study iPod-Touch.

Data from the mobile application was collected using a custom developed web server (Golang Language, RethinkDB). Data transfer was designed to be actively initiated by the study participants with all data being encrypted before transfer to the central server and transferred using SSL/TLS secured connections.

1. **Interactive Paper-Prototypes: POP** [https://marvelapp.com/pop]
2. **Mobile App Design: Sketch** [https://www.sketchapp.com]
3. **Interactive Design-Prototypes: Invision** [https://www.invisionapp.com]
4. **Mobile App Development: Swift 3** [https://developer.apple.com/swift]
5. **Mobile App Development: Xcode 8.2** [https://developer.apple.com/xcode]
6. **Mobile App Development: Realm Mobile Database Library for data storage** [https://realm.io]
