# Supplementary material for: Validity of mobile electronic data capture in clinical studies: a pilot study in a pediatric population
Source: BMC Med Res Methodol. 2017 Dec 8;17:163. doi: 10.1186/s12874-017-0438-x (PMC5721383; doi:10.1186/s12874-017-0438-x)
Supplement: Supplementary file 2 — Timing of complete data and samples, by timepoint and location. Table S2. Complete and correct data and samples when timing as a correctness factor is neglected (i.e. all data collected within one calendar day are correct). Table S3. Caregiver feasibility questionnaire. Table S4. Main difficulties experienced by caregivers during conduct of study. (PDF 205 kb) [file 12874_2017_438_MOESM2_ESM.pdf]

Table 1. Timing of complete data and samples, by timepoint and location

			Timing correct			
			No		Yes	
**Location**	**Item**	**Total n**	**n**	**%**	**n**	**%**
At home	Pain scale tM	47	0	0.0	47	100.0
	Pain scale t1	47	18	38.3	29	61.7
	Saliva sample t1	41	16	39.0	25	61.0
	Pain scale t2	47	13	27.7	34	72.3
	Saliva sample t2	38	6	15.8	32	84.2
	All	220	53	24.1	167	75.9
Inpatient	Pain scale tM	47	0	0.0	47	100.0
	Pain scale t1	47	27	57.4	20	42.6
	Saliva sample t1	38	20	52.6	18	47.4
	Pain scale t2	47	21	44.7	26	55.3
	Saliva sample t2	39	17	43.6	22	56.4
	All	218	85	39.0	133	61.0
All	Pain scale tM	94	0	0.0	94	100.0
	Pain scale t1	94	45	47.9	49	52.1
	Saliva sample t1	79	36	45.6	43	54.4
	Pain scale t2	94	34	36.2	60	63.8
	Saliva sample t2	77	23	29.9	54	70.1
	All	438	138	31.5	300	68.5

Table 2. Complete and correct data and samples when timing as a correctness factor is neglected (i.e. all data collected within one calendar day are correct)

			Complete and correct			
			No		Yes	
**Location**	**Item**	**Total n**	**n**		**n**	**%**
At home	Pain scale	153	12	7.8	141	92.2
	Saliva sample	102	26	25.5	76	74.5
	All	255	38	14.9	217	85.1
Inpatient	Pain scale	150	9	6.0	141	94.0
	Saliva sample	100	27	27.0	73	73.0
	All	250	36	14.4	214	85.6
All	pain scale	303	21	6.9	282	93.1
	Saliva sample	202	53	26.2	149	73.8
	All	505	74	14.7	431	85.3

Table 3. Caregiver feasibility questionnaire

	n	%
What statement(s) apply in your opinion? (n=15)		
I think clinical studies at home are a good idea	9	60.0
I don't think clinical studies at home are a good idea	1	6.7
I am not sure	5	33.3
How were the aims of the study explained to you? (n=15)		
Very well	9	60.0
Well	5	33.3
Sufficiently	1	6.7
Not well	0	0.0
Not well at all	0	0.0
How were your tasks during the conduct of the study study explained to you? (n=15)		
Very well	4	26.7
Well	8	53.3
Sufficiently	3	20.0
Not well	0	0.0
Not well at all	0	0.0
How user-friendly do you rate the mobile application? (n=15)		
Great	6	40.0
Ok	8	53.3
Unusable	1	6.7
Who has mainly collected data and samples in the hospital? (n=16)		
Mother	9	56.2
Father	1	6.2
Both parents	3	18.8
Study Nurse	3	18.8
Child	0	0.0
Other	0	0.0
Who has mainly collected data and samples in at home? (n=15)		
Mother	11	73.3
Father	1	6.7
Both parents	3	20.0
Study Nurse	0	0.0
Child	0	0.0
Other	0	0.0
How feasible were the study procedures for you in the hospital? (ntot=15)		
Very easy	4	26.6
Easy	10	66.7
Difficult	1	6.7
Very difficult	0	0.0
How feasible were the study procedures for you at home? (n=15)		
Very easy	2	13.3

Easy	6	40.0
Difficult	7	46.7
Very difficult	0	0.0
How feasible was taking photos of concomitant medication for you? (n=15)		
Not used	4	26.7
Very easy	4	26.7
Easy	5	33.2
Difficult	1	6.7
Very difficult	1	6.7
How much time did you spend on study procedures per day? (n=15)		
Less than 15 minutes	10	66.7
15-30 min	4	26.6
30-45 min	1	6.7
45-60 min	0	0.0
more than 60min	0	0.0
Would you take part again in such a study? (n=15)		
Yes, for sure	4	26.7
Probably yes	8	53.3
Probably not	3	18.8
Definitely not	0	0.0

Table 4. Main difficulties experienced by caregivers during conduct of study

What were the main difficulties you experienced during the conduct of the study? (multiple choice)		
	**n**	**%**
It was sometimes difficult to follow the study procedures (i.e. timing of data collection)	13	86.7
There were technical issues with the mobile application	3	20.0
My child did not want to participate, i.e. provide saliva samples	3	20.0
I was not able to contact the study personnel	2	13.3
It took too much time to follow the study procedures	1	6.7
I did not experience any difficulties	1	6.7
Other	3	20.0
