# Supplementary figures and images for: Validity of mobile electronic data capture in clinical studies: a pilot study in a pediatric population
Source: BMC Med Res Methodol. 2017 Dec 8;17:163. doi: 10.1186/s12874-017-0438-x (PMC5721383; doi:10.1186/s12874-017-0438-x)

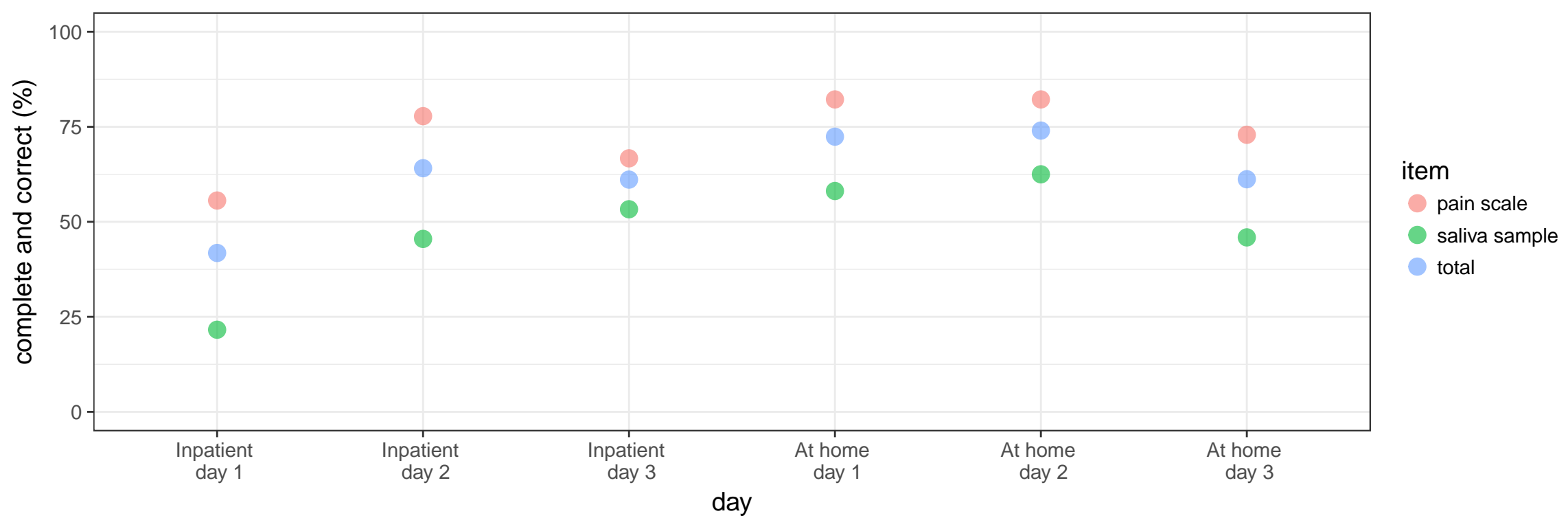

Supplement: Supplementary file 3 — Proportion of complete and correct data and samples by location and day. (PDF 6 kb) [file 12874_2017_438_MOESM3_ESM.pdf]

complete and correct (%)

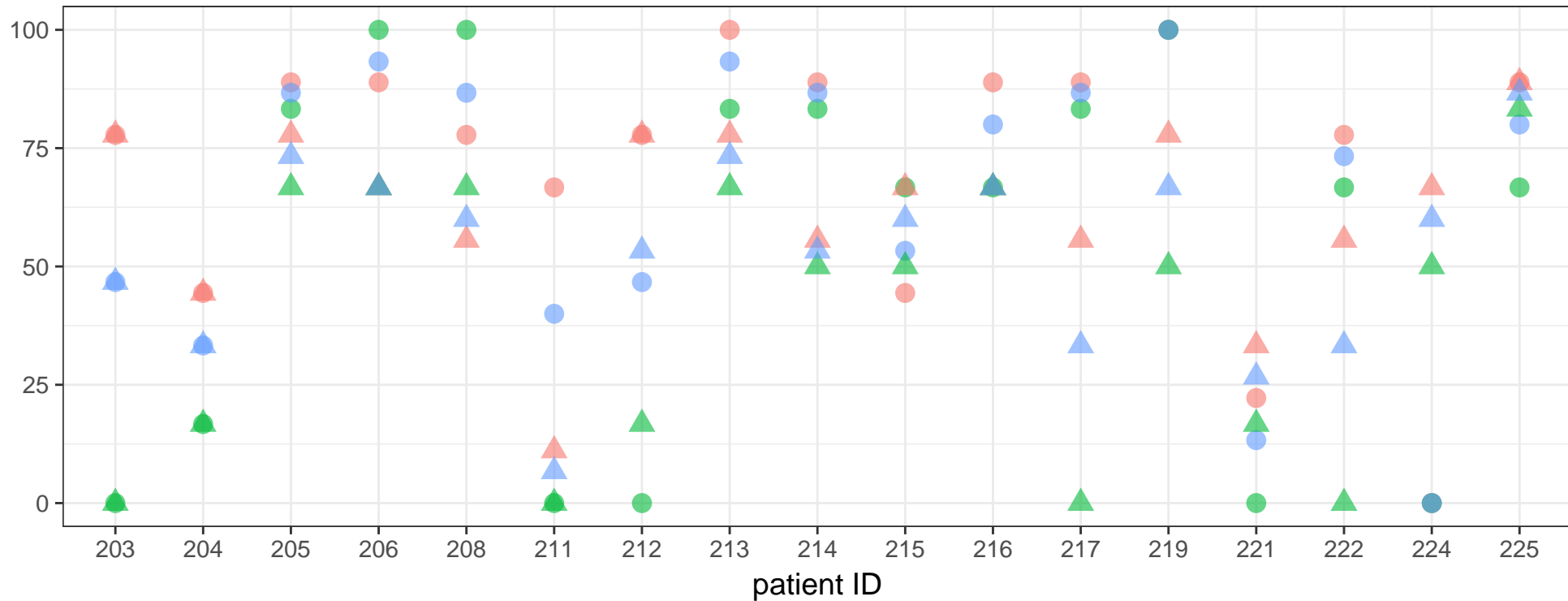

location

At home  
Inpatient

item

pain scale  
saliva sample  
total

Supplement: Supplementary file 4 — Proportion of complete and correct data and samples by patient and location. (PDF 8 kb) [file 12874_2017_438_MOESM4_ESM.pdf]
